# Clinical significance of t(11;14) translocation in systemic AL amyloidosis in the era of daratumumab therapy

**DOI:** 10.1007/s10238-025-02005-2

**Published:** 2025-12-22

**Authors:** Nao Nishimura, Yawara Kawano, Jun-ichirou Yasunaga

**Affiliations:** Department of Hematology, Rheumatology, and Infectious Diseases, Graduate School of Medical Sciences, Kumamoto University, 1-1-1 Honjo, Chuo-ku, Kumamoto, 860–8556 Japan

**Keywords:** AL amyloidosis, t(11;14), CD38, Daratumumab

## Abstract

The chromosomal translocation t(11;14)(q13;q32) is frequently observed in systemic light-chain (AL) amyloidosis, yet its clinical significance in the era of daratumumab-based therapy remains unclear. To compare clinical and cytogenetic characteristics, we retrospectively analyzed 68 patients with systemic AL amyloidosis and 107 patients with multiple myeloma (MM) newly diagnosed at Kumamoto University Hospital, with the MM cohort serving as a reference cohort. t(11;14) was detected in 55.9% of AL amyloidosis and 28.0% of MM cases. In both diseases, t(11;14) was associated with light chain–only M-protein and elevated CD20 expression on bone marrow plasma cells, suggesting a distinct phenotype. Notably, t(11;14)-positive AL amyloidosis patients exhibited a significantly lower incidence of renal dysfunction, a feature not observed in t(11;14)-positive MM. Among 47 AL amyloidosis patients treated with upfront daratumumab-containing regimens, overall survival did not differ significantly by t(11;14) status. However, event-free survival was significantly shorter in the t(11;14)-positive group (median 41.6 vs. 71.3 months, *p* = 0.037), accompanied by inferior 3 months hematological and cardiac responses. These findings suggest that t(11;14)-positive AL amyloidosis constitutes a distinct biological and clinical subtype characterized by delayed treatment response to daratumumab. Tailored therapeutic strategies targeting the unique biology of t(11;14)-positive AL amyloidosis is warranted.

## Introduction

Immunoglobulin light-chain amyloidosis (AL amyloidosis) is a monoclonal plasma cell disorder in which monoclonal immunoglobulin light-chain-derived amyloid fibrils deposit throughout the body, leading to organ failure. Patients with cardiac involvement in AL amyloidosis have been considered to have poor prognosis, which led to the attempt to develop more effective treatments. The introduction of daratumumab, a CD38 monoclonal antibody, has profoundly impacted the outcomes of AL amyloidosis patients [1–3]. However, treatments against patients with severe organ disfunctions and patients refractory to daratumumab based therapies are still challenging. Moreover, the biological features of monoclonal plasma cells and the mechanisms underlying the development of AL amyloidosis remain largely unclear.

The chromosomal translocation t(11;14)(q13;q32) in bone marrow monoclonal plasma cells (BMPCs), which brings together the immunoglobulin heavy-chain locus and the oncogene cyclin D1, occurs in approximately 40–60% of AL amyloidosis cases [4, 5] and 15–20% of multiple myeloma (MM) cases [6, 7]. Several common features have been reported in t(11;14) positive MM cases such as higher incidences of specific M protein subtypes (light chain only, immunoglobulin (Ig) M, IgE, and non-secretory) [6, 8] and unique surface molecule expression patterns (higher CD20 positivity and decreased CD56 expression) of BMPCs [9]. Furthermore, t(11;14) positivity is generally considered as a standard-risk feature with neutral prognosis in MM [10] while in AL amyloidosis, the presence of t(11;14) is linked to inferior response to bortezomib-based regimens [4, 11].

Although the frequency of t(11;14) positive cases are much higher in AL amyloidosis compared to MM, analysis of its’ baseline characteristics is lacking. Moreover, the clinical impact of t(11;14) positivity in AL amyloidosis patients receiving upfront daratumumab therapies are still a matter of debate. This single-center retrospective study analyzed the clinical characteristics of AL amyloidosis cases with or without t(11;14) BMPCs and were compared with MM cases. Additionally, differences in therapeutic response and survival of upfront daratumumab based therapies in newly diagnosed AL amyloidosis patients were investigated regarding the presence of t(11;14).

## Materials and methods

### Patients and study design

Baseline characteristics according to t(11;14) status of 68 patients with systemic AL amyloidosis and 107 patients with MM, newly diagnosed at Kumamoto University Hospital between 2013 and 2024 with available fluorescence in situ hybridization (FISH) data, were analyzed. All patients were diagnosed with systemic AL amyloidosis or MM according to the International Myeloma Working Group (IMWG) criteria [12]. Disease staging was defined by the Mayo 2012 criteria for AL amyloidosis [13] and the International Staging System (ISS) for MM [14]. Flow cytometric analysis of BMPCs were performed by the commercially available CD38 multi-analysis (SRL Laboratories, Tokyo, Japan) by gating CD38 highly positive fractions as described previously [15]. The cell surface expression of CD20 and CD56 was considered positive when more than 20% of CD38 high BMPCs expressed the respective markers [16]. Overall survival (OS), event free survival (EFS), hematological and cardiac response of systemic AL amyloidosis patients treated with upfront daratumumab containing regimens were retrospectively analyzed. The study was conducted in accordance with the principles of the Declaration of Helsinki and was approved by the Institutional Review Board and Ethics Committee of Kumamoto University.

### Response assessment and survival

The OS and EFS of 47 systemic AL amyloidosis patients treated with upfront daratumumab containing regimens (i.e. daratumumab, cyclophosphamide, bortezomib and dexamethasone (DCyBorD)(*n* = 18) [1] or daratumumab, lenalidomide and dexamethasone (DRD)(*n* = 29) [2]) were analyzed. EFS was determined as survival free from major organ deterioration (end-stage cardiac or renal failure) or hematologic progression according to the ANDROMEDA trial [1]. Hematologic response, 3 months and 6 months post daratumumab therapy according to t(11;14) status, was determined according to AL amyloidosis hematological response criteria using difference between serum involved and uninvolved free light chain (dFLC) [17]. Cardiac response of patients with cardiac amyloidosis was evaluated at 3- and 6-months post treatment. Brain natriuretic peptide (BNP)-based criteria [18, 19] was used for analyzing cardiac response, due to limited access to N-terminal probrain natriuretic peptide (NT-proBNP) at Kumamoto University Hospital.

### Statistical analysis

Baseline characteristics of the patients were compared using the Mann-Whitney U-test for continuous variables and the chi-square test for categorical variables. OS and EFS were estimated using the Kaplan–Meier method, and differences between groups were assessed by the log-rank test. P values less than 0.05 were considered statistically significant. Statistical analysis was performed with GraphPad Prism software (Boston, MA).

## Results

We first compared the baseline clinical and cytogenetic characteristics between patients with AL amyloidosis and those with MM (Table 1). The median age was comparable between the AL and MM cohorts (69.3 vs. 69.3 years). Compared with MM, patients with AL amyloidosis exhibited significantly higher frequencies of light chain–only M protein (66.2% vs. 24.3%, *p* < 0.0001), λ light chain isotype (80.9% vs. 30.8%, *p* < 0.0001), and the translocation t(11;14) (55.9% vs. 28.0%, *p* = 0.0002).

**Table 1:** Baseline characteristics of the patients enrolled in the study

	AL (*n* = 68)	MM (*n* = 107)	*p* value
Median age (range)	69.3 (42.3–86.0)	69.3 (32.7–85.4)	0.81
Male-no (%)	43 (63.2)	57 (53.3)	0.092
Heavy chain isotype-no (%)			
None	45 (66.2)	26 (24.3)	***< 0.0001***
IgG	14 (20.6)	58 (54.2)	***< 0.0001***
IgA	6 (8.8)	19 (17.8)	0.1
Light chain isotype-no (%)			
λ	55 (80.9)	33 (30.8)	***< 0.0001***
Cytogenic abnormality-no (%)			
t(11;14) (+)	38 (55.9)	30 (28.0)	***0.0002***
BMPC phenotype-no (%)			
CD20+	11 (16.2)	13 (12.1)	0.45
CD56+	41 (60.3)	74 (69.2)	0.23

Statisticaly significant (*p*<0.05) values are indicated in bold italic

AL, immunoglobulin light-chain amyloidosis; MM, multiple myeloma; BMPC, bone marrow monoclonal plasma cells

MM patients with t(11;14) (*n* = 30) exhibited a significantly higher frequency of light chain–only M protein (50.0% vs. 14.3%, *p* = 0.0001) and a lower incidence of IgG-type M protein (26.7% vs. 64.9%, *p* = 0.0004) compared with those without the translocation (Table 2). CD20 expression on BMPCs was markedly more frequent in t(11;14)-positive MM (33.3% vs. 3.9%, *p* < 0.0001), whereas CD56 positivity was significantly reduced (46.7% vs. 77.9%, *p* = 0.0017). In contrast, there were no significant differences between the two groups regarding renal function, anemia, bone lesions, or ISS stage.

In AL amyloidosis, patients harboring the t(11;14) translocation (*n* = 38) demonstrated a significantly higher prevalence of light-chain–only M-protein (84.2% vs. 43.4%, *p* = 0.0004), a lower frequency of the IgA isotype (0% vs. 20%, *p* = 0.0039), and more frequent CD20 expression on BMPCs (28.9% vs. 0.0%, *p* = 0.0013) compared with t(11;14)–negative cases (Table 2). In contrast, renal dysfunction defined as serum creatinine ≥ 2.0 mg/dL was less common in the t(11;14)-positive group (0.0% vs. 26.7%, *p* = 0.0007). No significant differences were observed in the proportion of Mayo 2012 stage IV cases or in the pattern of organ involvement by amyloid deposition.

**Table 2:** Clinical characteristics according to t(11;14) positivity in AL amyloidosis and MM

MM			
	t(11;14) (+) (*n* = 30)	t(11;14) (-) (*n* = 77)	p value
Median age (range)	69.1 (32.2–81.0)	69.6 (32.7–85.4)	0.64
Male-no (%)	17 (56.7)	40 (51.9)	0.66
Heavy chain isotype-no (%)			
None	15 (50.0)	11 (14.3)	***0.0001***
IgG	8 (26.7)	50 (64.9)	***0.0004***
IgA	3 (10.0)	16 (20.6)	0.19
λ Light chain isotype-no (%)	11 (36.7)	22 (28.6)	0.42
CD20 + BMPC-no (%)	10 (33.3)	3 (3.9)	***< 0.0001***
CD56 + BMPC-no (%)	14 (46.7)	60 (77.9)	***0.0017***
ISS stage Ⅲ-no (%)	12 (40.0)	26 (33.8)	0.55
Crea ≧ 2.0 mg/dL-no (%)	7 (23.3)	11 (14.3)	0.26
Hgb ≦ 10 g/dL-no (%)	14 (46.7)	36 (46.8)	0.99
Bone disease-no (%)	20 (66.7)	53 (68.8)	0.83

AL			
	t(11;14) (+) (*n* = 38)	t(11;14) (-) (*n* = 30)	p value
Median age (range)	69.3 (42.4–83.4)	68.6 (45.8–86.1)	0.77
Male-no (%)	25 (65.8)	18 (60)	0.62
Heavy chain isotype-no (%)			
None	32 (84.2)	13 (43.4)	***0.0004***
IgG	6 (15.8)	8 (26.7)	0.27
IgA	0 (0)	6 (20.0)	***0.0039***
λ light chain isotype-no (%)	30 (78.9)	25 (83.3)	0.65
CD20 + BMPC-no (%)	11 (28.9)	0 (0)	***0.0013***
CD56 + BMPC-no (%)	24 (63.2)	17 (56.7)	0.59
Mayo 2012 stage Ⅳ-no (%)	11 (28.9)	8 (26.7)	0.84
Crea ≧ 2.0 mg/dL-no (%)	0 (0)	8 (26.7)	***0.0007***
Involved organs -no (%)			
Heart	29 (76.3)	24 (80.0)	0.72
Kidney	22 (57.9)	14 (46.7)	0.36

Statistically significant values (*p*<0.05) are indicated in bold italics

MM, multiple myeloma; AL, immunoglobulin light-chain amyloidosis; BMPC, bone marrow monoclonal plasma cells; ISS, International Staging System; Mayo 2012 stage, Mayo 2012 staging system for AL amyloidosis; Crea, Serum creatinine; Hgb, hemoglobin.

**Fig. 1:**
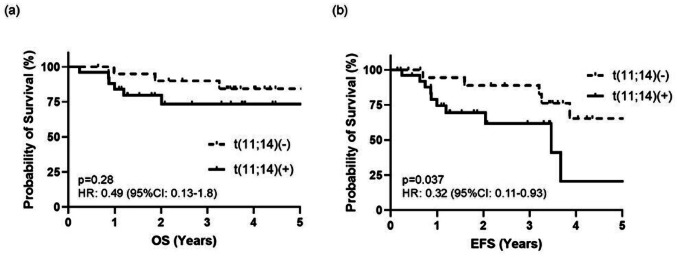
Survival of AL amyloidosis patients treated with upfront daratumumab based regimens. (**a**) OS (**b**) EFS

The OS and EFS of patients with AL amyloidosis treated with upfront daratumumab-containing regimens (*n* = 47) were analyzed according to the presence of the t(11;14) translocation (median follow-up, 41 months) (Fig. 1). Although OS did not differ significantly between patients with and without t(11;14) (hazard ratio (HR), 0.49; 95% confidence interval (CI), 0.13–1.8; *p* = 0.28), EFS was significantly shorter in the t(11;14)-positive group (median EFS, 41.6 vs. 71.3 months; HR, 0.32; 95% CI, 0.11–0.93; *p* = 0.037).

**Fig. 2:**
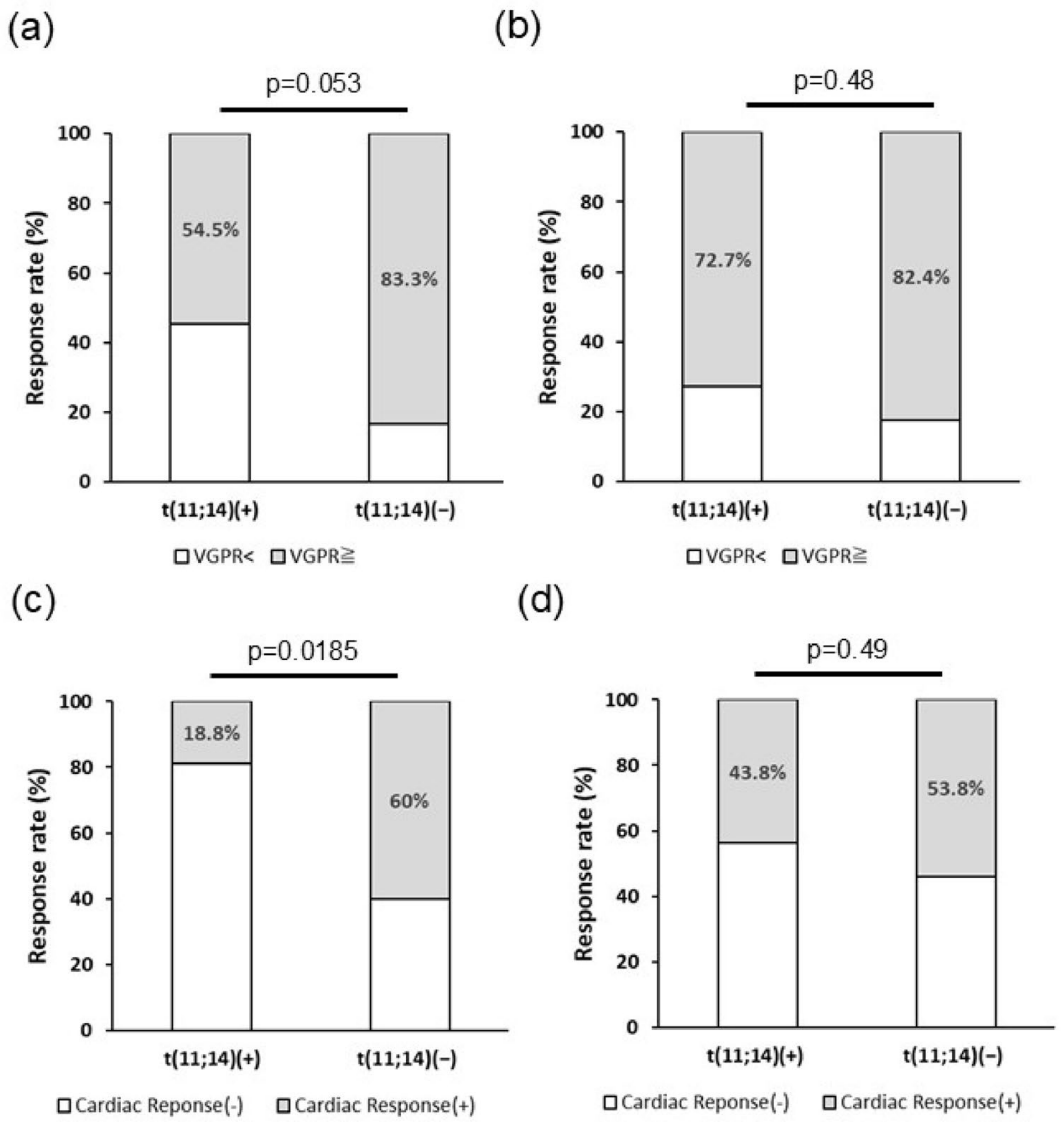
Therapeutic response of AL amyloidosis patients under daratumumab treatment. Hematological response 3 months (**a**) and 6 months (**b**) post daratumumab therapy. Cardiac response 3 months (**c**) and 6 months (**d**) post daratumumab therapy

To investigate the cause of the difference in EFS according to t(11;14) status, hematologic and cardiac responses were assessed 3 and 6 months after treatment initiation (Fig. 2). Although not statistically significant, t(11;14)-positive patients tended to exhibit lower hematologic response rates at 3 months compared with t(11;14)-negative patients (54.5% vs. 83.3%, *p* = 0.053), whereas the difference was no longer evident at 6 months (72.7% vs. 82.4%, *p* = 0.48). In contrast, t(11;14)-positive cardiac amyloidosis patients demonstrated a significantly lower cardiac response rate than t(11;14)-negative cases at 3 months following daratumumab-based therapy (18.8% vs. 60.0%, *p* = 0.019), though this difference was not observed at 6 months (43.8% vs. 53.8%, *p* = 0.49).

## Discussion

In the current study, we demonstrated that the t(11;14) translocation is strongly associated with distinct biological and clinical features in AL amyloidosis. The relatively higher prevalence of t(11;14) in our MM cohort compared with previous reports [7, 8, 10] is likely attributable to the small sample size of the study. Consistent with previous reports, t(11;14)-positive MM cases exhibited a predominance of light chain–only M-protein and higher CD20 expression on BMPCs [6, 8], suggesting a distinct plasma cell phenotype. A similar phenotype was also observed in t(11;14)-positive AL amyloidosis, underscoring the shared biological features of these entities. Although the evolutionary trajectory of t(11;14)-positive AL amyloidosis plasma cells remain incompletely understood, previous work has demonstrated that the t(11;14) translocation in MM can arise at the pro-B-cell stage [20]. This early oncogenic event may, at least in part, account for the “B-cell–like” phenotypic features—such as CD20 expression [6, 8] and lymphoplasmacytic morphology [21, 22] —frequently observed in t(11;14)-positive MM and potentially in AL plasma cells as well. In contrast, the M protein isotype pattern differed between MM and AL amyloidosis; t(11;14) was associated with fewer IgG-type M-proteins in MM, whereas in AL amyloidosis, the IgA-type was notably reduced. Furthermore, CD56 downregulation, a hallmark of t(11;14)-positive MM [9], was not evident in AL amyloidosis, suggesting disease-specific differences in plasma cell phenotype. CD56 expression on MM plasma cells has been implicated in enhanced cell survival and adhesion to the bone marrow stromal cells [23]. The differential pattern of CD56 expression observed between t(11;14)-positive MM and t(11;14)-positive AL plasma cells may reflect distinct biological adaptations to the bone marrow microenvironment; however, further detailed mechanistic analyses are warranted. Clinically, t(11;14)-positive AL amyloidosis patients exhibited a significantly lower incidence of renal dysfunction, consistent with previous findings [24]. The underlying mechanisms remain unclear but may reflect differences in amyloid fibril composition or organ tropism associated with the t(11;14) genotype.

Although OS was not significantly affected, t(11;14)-positive cases showed shorter EFS, potentially due to lower hematologic and cardiac responses during the early phase of daratumumab-based therapy, suggesting that clonal plasma cells harboring t(11;14) may display reduced sensitivity to CD38-targeted therapy or slower kinetics of clonal eradication.

The prognostic impact of t(11;14) in AL amyloidosis has shifted in the era of novel agents. Whereas bortezomib-based regimens previously yielded poor responses in this subgroup [4, 11], daratumumab appears to mitigate—but not fully eliminate—this adverse effect. Given the overexpression of BCL2 in t(11;14)-positive plasma cells, integration of BCL2 inhibitors such as venetoclax may offer therapeutic benefit [25]. A recent study demonstrated that AL plasma cells broadly exhibit upregulation of BCL-2 family members, and that t(11;14)-positive AL plasma cells display a relatively higher BCL2/BCL2L1 ratio compared with t(11;14)-negative cases [26]. These findings, together with the reported clinical efficacy of venetoclax in AL amyloidosis [25], support a biological rationale for BCL-2 inhibition in t(11;14)-positive AL patients. Nevertheless, the optimal timing, patient selection, and treatment context for BCL-2–targeted therapy in AL amyloidosis remains unresolved. Furthermore, emerging BCMA-targeted modalities, including bispecific antibodies, antibody–drug conjugates, and CAR-T cell therapies [27], hold promise for improving outcomes in this distinct molecular subset.

This study has several limitations. It was a retrospective analysis conducted at a single institution with a relatively small cohort, and treatment heterogeneity existed among patients receiving daratumumab-containing regimens (e.g., DCyBorD, DRD). These factors may have introduced selection and response biases and limit the generalizability of our findings. Therefore, larger multicenter, prospective studies are warranted to validate these observations.

Collectively, our findings reinforce that t(11;14)-positive AL amyloidosis constitutes a distinct molecular and clinical entity. Tailored therapeutic approaches that leverage its unique biological characteristics will be crucial for further improving patient outcomes in the daratumumab era.

## Data Availability

The data related to this report will be made available from the corresponding author upon reasonable request.
